# Methylselenol Formed by Spontaneous Methylation of Selenide Is a Superior Selenium Substrate to the Thioredoxin and Glutaredoxin Systems

**DOI:** 10.1371/journal.pone.0050727

**Published:** 2012-11-30

**Authors:** Aristi P. Fernandes, Marita Wallenberg, Valentina Gandin, Sougat Misra, Francesco Tisato, Cristina Marzano, Maria Pia Rigobello, Sushil Kumar, Mikael Björnstedt

**Affiliations:** 1 Division of Pathology F46, Department of Laboratory Medicine, Karolinska Institutet, Karolinska University Hospital Huddinge, Stockholm, Sweden; 2 Department of Pharmaceutical Sciences, University of Padova, Padova, Italy; 3 Corso Stati Uniti, 4, ICIS-CNR, Padova, Italy; 4 Department of Biological Chemistry, University of Padova, Padova, Italy; 5 Division of Environmental Carcinogenesis, CSIR-Indian Institute of Toxicology Research, Lucknow, India; Instituto de Biociencias - Universidade de São Paulo, Brazil

## Abstract

Naturally occurring selenium compounds like selenite and selenodiglutathione are metabolized to selenide in plants and animals. This highly reactive form of selenium can undergo methylation and form monomethylated and multimethylated species. These redox active selenium metabolites are of particular biological and pharmacological interest since they are potent inducers of apoptosis in cancer cells. The mammalian thioredoxin and glutaredoxin systems efficiently reduce selenite and selenodiglutathione to selenide. The reactions are non-stoichiometric aerobically due to redox cycling of selenide with oxygen and thiols. Using LDI-MS, we identified that the addition of S-adenosylmethionine (SAM) to the reactions formed methylselenol. This metabolite was a superior substrate to both the thioredoxin and glutaredoxin systems increasing the velocities of the nonstoichiometric redox cycles three-fold. *In vitro* cell experiments demonstrated that the presence of SAM increased the cytotoxicity of selenite and selenodiglutathione, which could neither be explained by altered selenium uptake nor impaired extra-cellular redox environment, previously shown to be highly important to selenite uptake and cytotoxicity. Our data suggest that selenide and SAM react spontaneously forming methylselenol, a highly nucleophilic and cytotoxic agent, with important physiological and pharmacological implications for the highly interesting anticancer effects of selenium.

## Introduction

**Figure 1:**
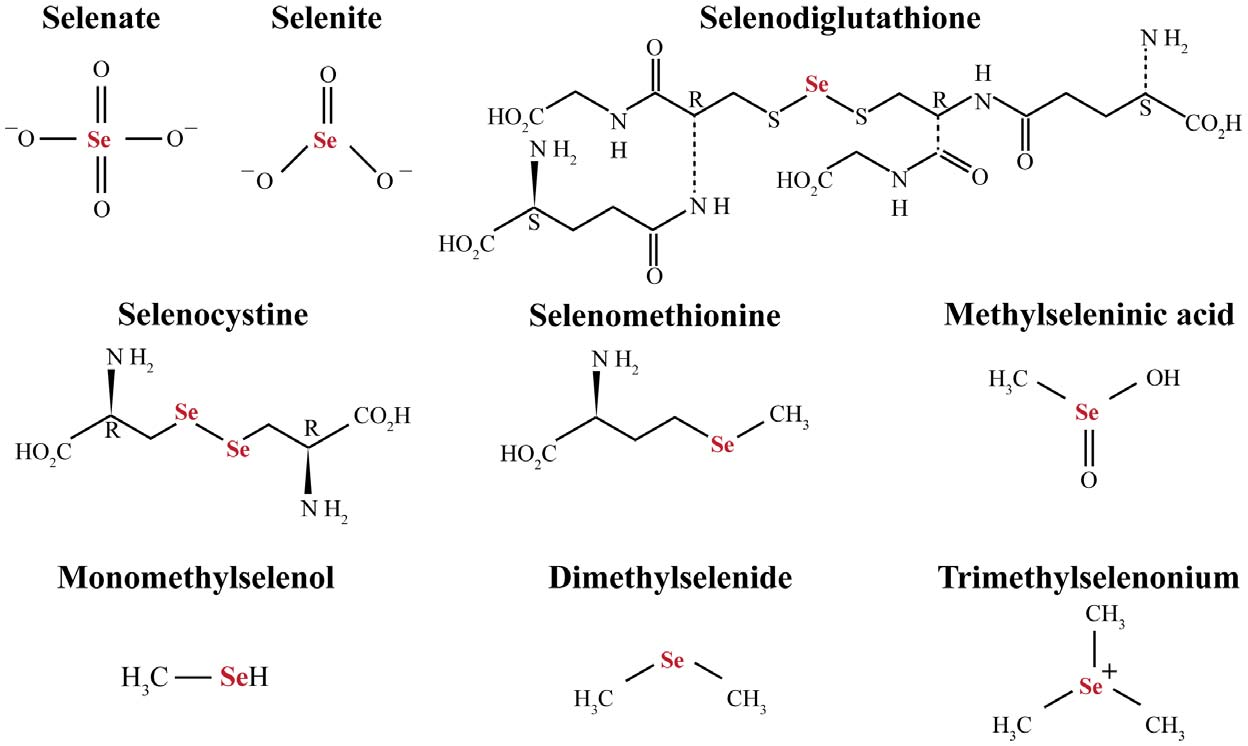
Structure of selenium compounds of interest in the present paper.

Selenium (Se) is an essential trace element in higher eukaryotes. One of the most established functions of organic selenium compounds in humans is their presence as selenocysteine residues in 25 different proteins, including the redox proteins glutathione peroxidase [1], 5′-iodothyronine deiodinase [2] and thioredoxin reductase (TrxR) [3]. Inorganic selenium compounds (e.g., selenite SeO_3_^2−^) are metabolized through reduction by glutathione (GSH) [4], the glutaredoxin (Grx) [5] or the thioredoxin (Trx) systems [6]. The thioredoxin and glutaredoxin systems are essential to preserve the intracellular redox balance via reduction of protein disulfides and glutathione mixed disulfides [7]. In reaction with reduced glutathione (GSH), inorganic selenium in the form of selenite forms a covalent adduct, selenodiglutathione (GS-Se-SG), which is further metabolized into selenide (HSe^−^) by the thioredoxin or glutaredoxin systems [8], [9]. In these reactions, the highly reactive selenide redox-cycles with oxygen and oxidizes NADPH, generating a massive non-stoichiometric reactive oxygen species (ROS) production [6]. Selenide may either transform to elemental selenium (Se°), or may undergo methylation, participate in biosynthesis and incorporation as selenocysteine in proteins [10], form selenosugars, and sequester metal ions [11], [12], [13]. In biological systems, intake of high doses of selenium compounds results in the generation of selenide followed by methylation to form methylselenol, dimethylselenide and trimethylselenonium [14], [15], [16]. The dimethylselenide (volatile form) and trimethylselenonium (non-volatile form) are the best known excretory metabolites of selenium in mammals [16]. In Figure 1, the different selenium compounds mentioned are summarized.

Being an essential trace element, selenium is known to have crucial roles in health and medicine. Low molecular compounds, like selenocystine, ebselen and diphenyl diselenide exhibiting peroxidase-like activity show medicinal importance and control bacterial infections, inflammatory reactions, ischemia and cancer [17], [18], [19], [20], [21]. In recent times, promising chemopreventive and chemotherapeutic potential of selenium compounds have been demonstrated [21], [22], [23], [24]. However, the difference between prevention and treatment are strictly dose dependent. The major mechanisms responsible for the efficacy exerted by the selenium compounds in cancer treatment are, instead, the massive ROS production and specific selenium uptake by tumor cells [21]. On the other hand, the mechanism behind selenium mediated chemoprevention has generally been addressed to incorporation of selenium in antioxidant proteins (e.g., GPx, TrxR) and their redox activity by maintaining the redox balance within the cells [25], [26], [27]. The continuous interest in medicinal role of selenium compounds can be viewed in reports on synthesis of different types of selenium containing compounds, with focus on their possible use in treatment of diseases including cancer, or for developing new and powerful antioxidants [28], [29]. Selenium metabolites like methylselenol and methylseleninic acid are believed to be the key intermediates conclusive for effective cancer prevention and treatment [23], [24]. Chemoprevention by methylselenol influences the adhesive and invasive properties of cancer cells by suppression of integrin expression [30], induction of caspase-mediated apoptosis [31], and influencing the silenced tumor suppressor proteins [32]. Methylselenol has also been reported to induce G1-cell cycle arrest and apoptosis via several cancer signaling genes [33].

The major methylation reactions in cells are mediated via S-adenosylmethionine (SAM), an important methyl group donor present in all cells. Methyl group from SAM is transferred to DNA, proteins, phospholipids and neurotransmitters in several metabolic pathways catalyzed by methyltransferase enzymes [34]. Through methylation cycle, SAM is also crucial for aminopropylation and trans-sulfuration by demethylation of SAM and formation of glutathione via homocysteine [35]. SAM has previously been proposed to be important in selenium metabolism and toxicity. In *Saccharomyces cerevisiae*, blockage of the pathway converting methionine to SAM resulted in increased incorporation of selenomethionine (SeMet) and decreased toxicity of this selenium compound [36].

In this study, we aimed to explore whether methylselenol may be spontaneously formed from selenide in the presence of SAM and thus provide an alternative mechanism for the pharmacological effects of selenium. The reactions of methylselenol with the thioredoxin and glutaredoxin systems and its toxicity were compared with other selenium compounds.

**Figure 2:**
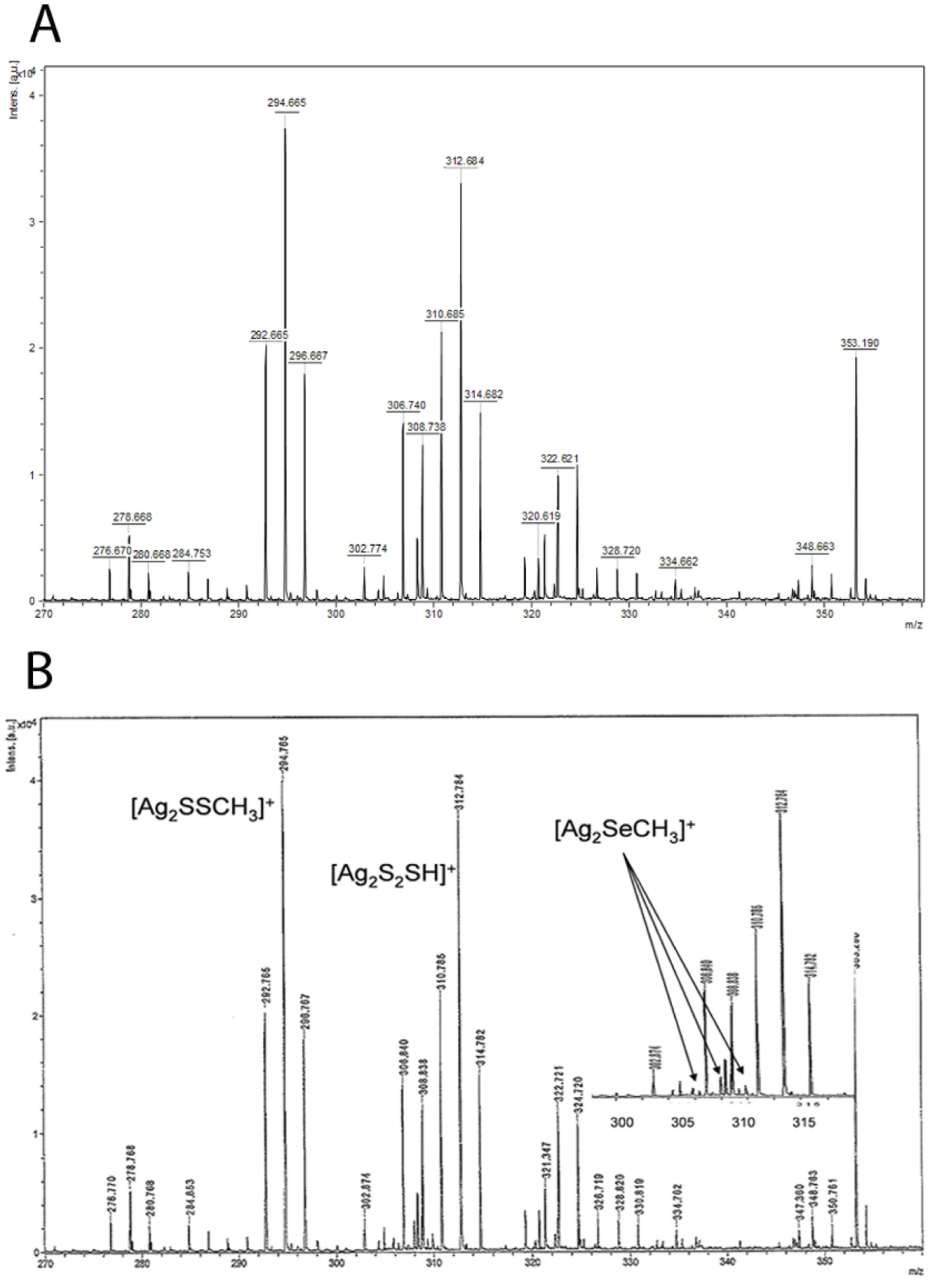
GC-Mass spectrum of methylselenol. Positive ion LDI-TOF mass spectrum in the 270–360 m/z region of the precipitated silver salts. **A**) In absence of Trx1, no peaks corresponding to [Ag_2_SeCH_3_]^+^ ion were detected. **B**) However, in the presence of Trx1, the spectrum shows the [Ag_2_SeCH_3_]^+^ ion (peaks in the range 304.8–314.8 m/z) overlapped with more abundant Ag_2_-containing clusters. In particular, low-intensity peaks at 305.8, 307.8 and 309.8 m/z (designated by arrows) denote the presence of selenium in the [Ag_2_SeCH_3_]^+^ molecule.

**Figure 3:**
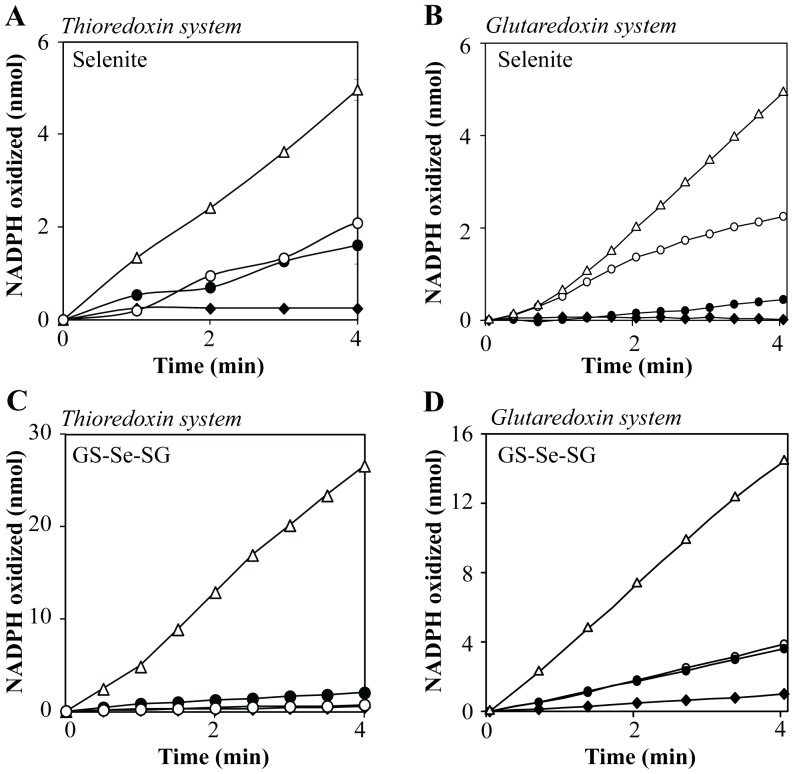
A–D. Oxidation of NADPH by the thioredoxin and glutaredoxin systems in the presence of selenite and GS-Se-SG, catalyzed by SAM. Reaction catalyzed by the thioredoxin system was performed in 50 mM Tris-HCl, 1 mM EDTA, pH 7.5, and 200 µM NADPH. A) The reaction mixture contained 2 µM human Trx1 and 50 nM mammalian TrxR1. SAM, 4 mM (♦) Selenite (5 µM) (○), SAM and selenite, 4 mM and 5 µM respectively (without Trx1 in the reaction) (•), SAM and selenite (Δ). B) Reaction catalyzed by the glutaredoxin system was performed in TE-buffer, containing 200 µM NADPH, 6 µg/mL GR, 1 µM human Grx1 and 50 µM GSH. SAM, 4 mM (♦) Selenite (5 µM) (○), SAM and selenite, 4 mM and 5 µM respectively (without Grx1 in the reaction) (•), SAM and selenite, 4 mM and 5 µM respectively (Δ). C) The thioredoxin system with: 5 µM GS-Se-SG (without Trx1 in the reaction) (♦), 5 µM GS-Se-SG (•), 5 µM GS-Se-SG and SAM (without Trx1 in the reaction) (○), 5 µM GS-Se-SG and SAM (Δ). D) The glutaredoxin system with: 15 µM GS-Se-SG (♦), GS-Se-SG and SAM (•), GS-Se-SG and 1 µM Grx1 (○), GS-Se-SG, SAM and 1 µM Grx1 (Δ).

## Experimental

### Chemicals

AgNO_3,_ BSA, DTNB, NADPH, insulin, monosodium glutamate (MSG), selenite, seleno-DL-cystine, methylseleninic acid (MSA), SAM, and tert-butyl hydroperoxide, were all purchased from Sigma Aldrich. Hydroethidine was obtained from Invitrogen and GS-Se-SG was purchased from PharmaSe. Mammalian thioredoxin reductase 1 (TrxR1), recombinant human Trx1 (Trx1C61S/C72S), *Escherichia coli* TrxR1, *Escherichia coli* Trx1 and human Grx1 were all purchased from IMCO Corporation.

### Methylselenol Production and Identification via Laser Desorption Ionization (LDI)-Mass Spectrometry (MS)

Selenols precipitates as silver-colored selenolates when passing through an aqueous silver nitrate solution. To verify the formation of methylselenolate, a method essentially as described by Gromer et al. [37] was used. In a freshly prepared mixture, containing degassed 50 mM Tris (pH 7.5), 1 mM EDTA, 200 µM NADPH, 100 nM *E. coli* TrxR1 and 2 µM *E. coli* Trx1, 5 µM selenite and 4 mM SAM were added to a final volume of 1 ml. The reaction was made anaerobic by uninterruptedly flushing with oxygen-free nitrogen (70 ml min^−1^). The reaction mixture was filtered through a spin column (Amicon Ultra, Millipore) with a cut off of 3 K. A yellow precipitate was formed by adding 0.1 M AgNO_3_ trapping solution to the reaction mixture. Nitrogen flushing was continued for another 15 min. The vial containing the precipitate was centrifuged at 10 000* g* and submitted to mass spectrometry. One microliter of sample solution (DMSO) was deposited on the stainless steel sample holder and allowed to dry before introduction into the mass spectrometer. The dried sample was analyzed by laser desorption/ionization (LDI) mass spectrometry. LDI mass spectrometric measurements were performed using a MALDI/TOF/TOF UltrafleXtreme instrument (Bruker Daltonics, Bremen, Germany), equipped with 1 kHz smartbeam II laser (λ = 355 nm) and operating in the positive reflectron modes.

**Table 1:** Peroxidase activity.

Condition	t-BHP* (nmol)
TrxR/Trx	0.40
TrxR/Trx/SAM	1.25
TrxR/Trx/Selenite	3.80
TrxR/Trx/SAM/Selenite	12.00

* The amount of t-BHP reduced was calculated from the amount NADPH oxidized after 6 min. All experiments were performed in TE-buffer with human Trx (2 µM), TrxR (50 nM), and NADPH (500 µM). Final concentration of selenite was 5 µM and 4 mM for SAM.

**Table 2:** Peroxidase activity.

Condition	t-BHP* (nmol)
Grx1/GSH	1.01
Grx1/GSH/SAM	1.11
Grx1/GSH/Selenite	12.58
Grx1/GSH/SAM/Selenite	32.75

* The amount of t-BHP reduced was calculated from the amount NADPH oxidized after 6 min. The mixture contained 50 µM GSH, 0.2 mM NADPH, 2 mM EDTA, 0.1 mg/mL BSA, and 6 µg/mL yGR and 1 µM Grx in 100 mM Tris-HCl. Selenite and SAM were added to a final concentration of 5 µM and 4 mM respectively.

**Figure 4:**
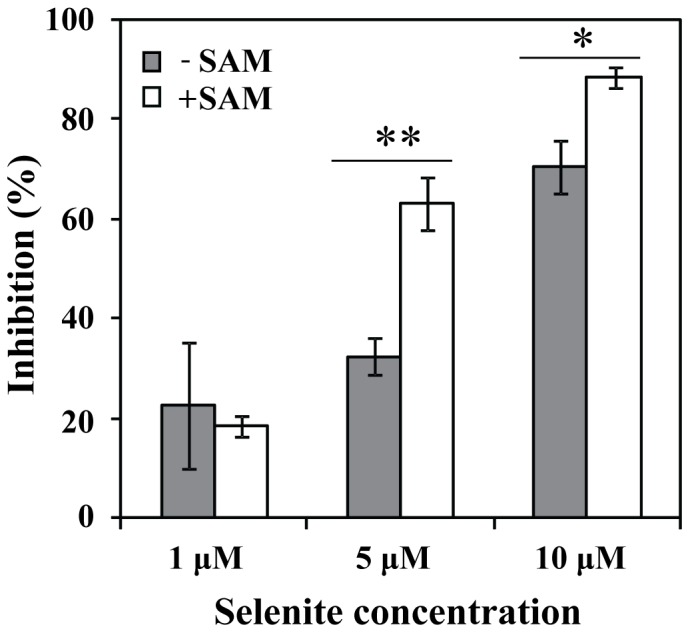
Inhibition of thioredoxin mediated protein disulfide reductase activity. The method was performed as described by Kumar et al. [6]. The reaction mixture contained 80 mM HEPES buffer, pH 7.6, 3 mM EDTA and 0.7 mM NADPH. TrxR1 and Trx1 were added to a final concentration of 8 nM and 1 µM respectively. The measurements were performed with the following selenite concentrations (1, 5 and 10 µM). The amount of SH-group formed was measured at 412 nm. Grey bars: Addition of selenite at varying concentrations. White bars: Addition of both selenite and SAM (4 mM). Student t-test, dependent by samples, was used for statistical analysis (*p<0.05, **p<0.01).

**Figure 5:**
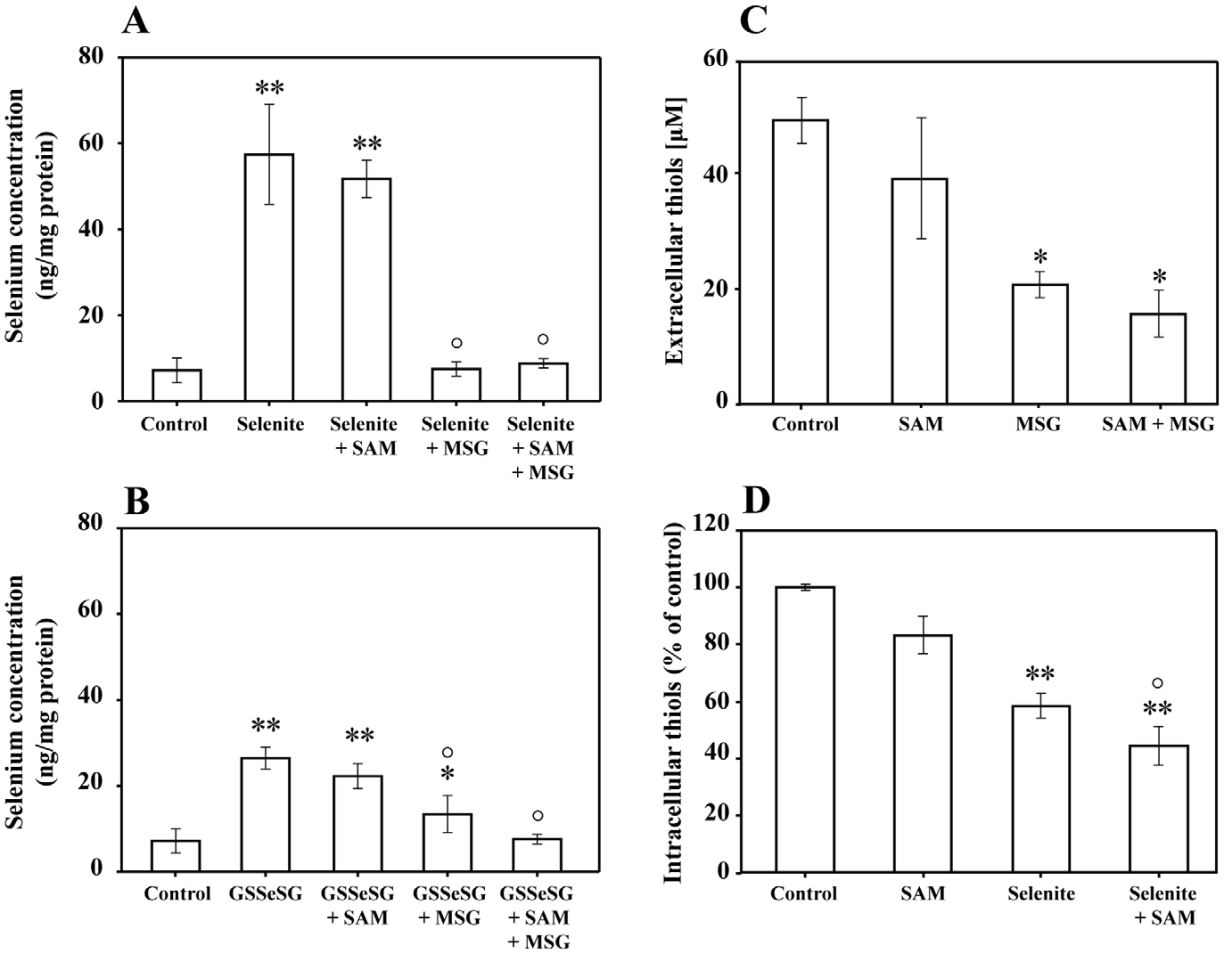
A–D. Total selenium accumulation, extracellular and intracellular thiols after treatment with various selenium compounds. Selenium accumulation in ng/mg protein after 5 h treatment with A) 5 µM selenite +/− SAM and MSG B) 5 µM GS-Se-SG +/− SAM and MSG measured by GF-AAS analysis. C) Total extracellular thiol content after 5 h treatment with 5 µM selenite +/− SAM and MSG. (*p<0.05 compared to control) and D) total intracellular thiol content following 5 h treatment with selenite (5 µM) +/− SAM was determined by the DTNB assay. Statistical analysis was performed by one-way ANOVA (95% confidence interval) followed by Tukey-Kramer multiple comparison test. (*p<0.05, **p<0.01 and ***p<0.001 compared to controls, °p<0.01 compared to selenium treated cells).

### Thioredoxin and Thioredoxin Reductase Activity Measurements

All experiments were performed in 50 mM Tris-HCl pH 7.5 containing 1 mM EDTA and 200 µM NADPH. The reactions were followed by NADPH consumption at A_340_ using the Ultrospec 4300 pro spectrophotometer (Amersham Biosciences) and activity was determined by the oxidation of NADPH using a molar extinction coefficient of 6200 M^−1^cm^−1^ as previously described [38].

**Figure 6:**
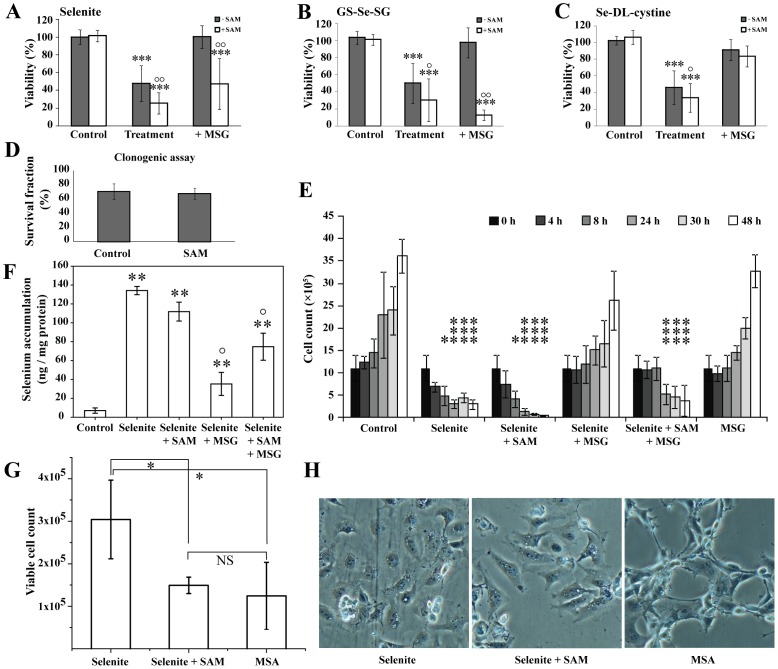
A–H. Cytotoxicity of selenium compounds in the presence of SAM. Cell viability was measured by XTT after 24 h incubation with selenium treatment, combined with SAM, DTNB and MSG. Cells were pretreated with MSG (60 mM) followed by treatment with selenium compounds +/− SAM (500 µM) **A**) Selenite (5 µM), **B**) GS-Se-SG (5 µM), **C**) Seleno-DL-cystine (100 µM). **D**) SAM toxicity was determined by clonogenic assay. Cells were treated for 8 h with 500 µM SAM, washed and re-seeded, in triplicates. After 9 days, clones were stained and counted. **E**) Viability over time (0–48 h) after pretreatment with MSG followed by addition of selenite +/− SAM. **F**) Selenium accumulation in ng/mg protein after 24 h treatment (same concentration of all compounds as in **E**) measured by GF-AAS analysis. **G**) Comparison of toxicity between selenite (5 µM) +/− SAM and MSA (5 µM) after 24 h of treatment. **H**) Representative morphological changes associated with the treatments of selenite (5 µM), selenite +/− SAM and MSA (5 µM) for 20 h. In **D**, Student t-test was performed to verify the statistical significance between two groups. One-way ANOVA (99.9% confidence interval) followed by Tukey-Kramer multiple comparison test was performed to determine statistical significance in **A–C, F** (**p<0.01 and ***p<0.001 compared to controls, °p<0.01 and °°p<0.001 compared to selenium treated cells). In **E**, two-way ANOVA (95% confidence interval) was performed, followed by Bonferroni multiple comparison test. (*p<0.05 and ***p<0.001, compared to control at selected time point). In Fig. **G**, one-way ANOVA was used, followed by Student-Newman-Keuls multiple comparison test (95% confidence interval, *p<0.05 compared to selenite treatment).

**Figure 7:**
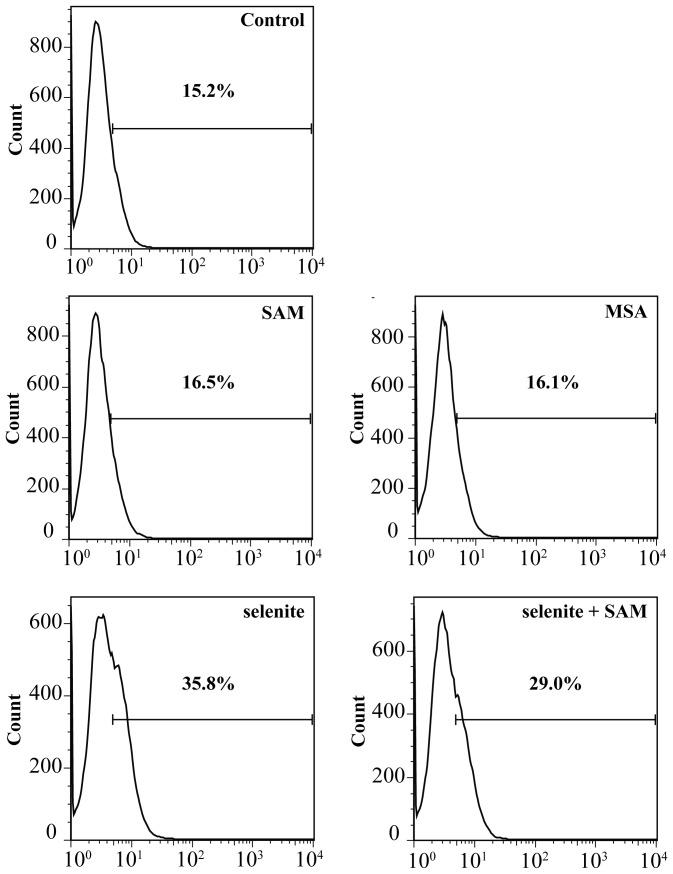
Effect of selenium compounds on superoxide production. H157 cells were stained with hydroethidine and treated for 5 h with selenite (5 µM) +/− SAM (500 µM), and MSA (5 µM) before detection of accumulated superoxide produced by FACS analysis, as described under materials and methods.

**Figure 8:**
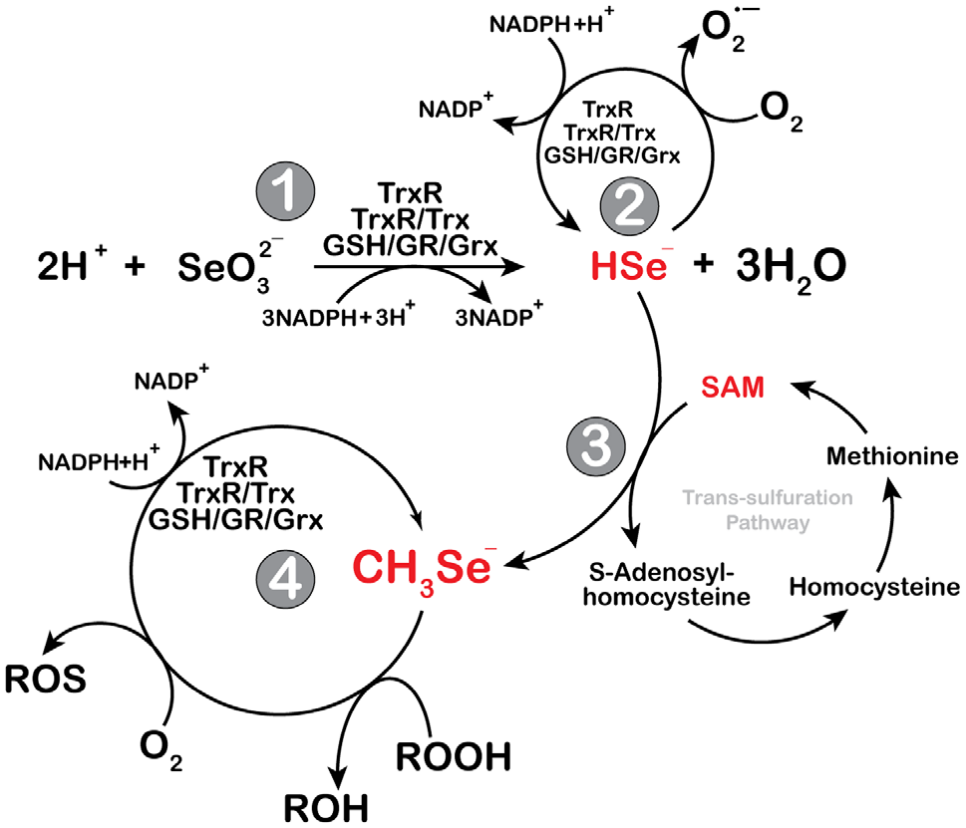
Proposed schematic overview of the spontaneous methylation of selenide to methylselenol. A schematic diagram showing the individual role of selenite, thioredoxin and glutaredoxin system and SAM in redox cycling of selenium intermediate metabolites. Selenite is reduced to hydrogen selenide either by thioredoxin or glutaredoxin system (reaction 1) [8], [9]. The same reaction can be catalyzed either by glutathione or cysteine, resulting into the same final end product. Hydrogen selenide can successively be oxidized by oxygen to form superoxide radical or undergo redox cycling mediated by thioredoxin or glutaredoxin system (reaction 2) [8], [9]. However, hydrogen selenide can spontaneously react with SAM to form methylselenol (reaction 3). Subsequently, the thioredoxin and glutaredoxin system participate in the redox cycling of methylselenol in a similar way (reaction 4) with that of hydrogen selenide and generate reactive oxygen species. Under reducing environment, monomethylselenol may act as a radical scavenger because of its superior nucleophilicity compared to its counterpart hydrogen selenide.

### Glutaredoxin Activity Measurements

To determine the reduction of selenium compounds by glutaredoxin together with SAM, a fresh mixture containing 1 mM GSH, 0.2 mM NADPH, 2 mM EDTA, 0.1 mg/ml BSA and 6 µg/ml yeast glutathione reductase in 100 mM Tris-HCl, pH 8.0 was prepared. The assay was performed in the same manner as previously described for the detection of the specific activity of glutaredoxins [39], but the method was adapted for use of a plate reader. Glutaredoxin activity, corresponding to µmols NADPH oxidized per minute, was calculated using the molar extinction coefficient of NADPH (ε = 6200 M^−1^cm^−1^), with a final reaction volume of 100 µl. In the reaction with selenite, the GSH content was reduced to 50 µM to minimize the background reduction and enable monitoring of the reaction. NADPH oxidation was monitored at A_340,_ using a Power Wave HT, Microplate Spectrophotometer from BioTek.

### Protein Disulfide Reductase Activity

The effects of selenite and SAM on the protein disulfide reductase activity of the human thioredoxin system were determined by the method previously described by Holmgren and Björnstedt [38]. Final concentrations of TrxR1, Trx1, selenite and SAM were 8 nM, 1 µM, 5 µM and 4 mM, respectively. The samples were incubated for 20 min at 25°C, and the reactions were stopped by the addition of DTNB in 6 M GuHCl and 0.2 M Tris-HCl. The amount of SH-groups formed was monitored at 412 nm.

### Cytochrome C reduction

The reduction of cytochrome C was performed with the same mixture as for the protein disulfide reductase activity, but with 50 nM TrxR1, 500 nM Trx1, 50 nM selenite and 4 mM SAM. The reaction mixture was pre-incubated with selenite for 10 minutes, followed by addition of SAM to the reaction and incubated for another 10 minutes. The reaction was initiated by addition of cytochrome C and monitored at 550 nm for 10 minutes. The amount of reduced cytochrome C was calculated using the molar extinction coefficient of reduced cytochrome C (ε = 28 mM^−1^cm^−1^).

### Cell Experiments

The well-established large cell lung carcinoma cell line, H157 (**ATCC**), was cultured in 1640 RPMI medium supplemented with 10% (v/v) fetal bovine serum, in 75 cm^2^ culture flasks (Sarstedts). Cells were grown in 37°C and 5% CO_2_. For the cell experiments, cells were treated with the specific compounds for different time points up to 48 h, depending on experimental methods that are described in detail below.

### Selenium Uptake

H157 cells (7.0×10^5^) were seeded in 25 cm^2^ flasks in growth medium (10 ml). After 20 h, the medium was replaced and the cells were incubated for 5 h in the presence of selenite and GS-Se-SG +/− SAM and MSG. Cells were washed with PBS and harvested. Cell pellets were subjected to three freezing/thawing cycles at −80°C, and then vigorously vortexed. Aliquots were removed for the determination of protein content by the BioRad protein assay kit (BioRad). The samples were added with 1 ml highly pure nitric acid (Se: ≤0.01 µg/kg, TraceSELECT® Ultra, Sigma Chemical Co.) and transferred into a microwave teflon vessel. Subsequently, samples were submitted to standard procedure using a speed wave MWS-3 Berghof instrument (Eningen, Germany). After cooling, each mineralized sample was analyzed for selenium content by using a Varian AA Duo graphite furnace atomic absorption spectrometer (Varian, Palo Alto, CA; USA) at the wavelength 196 nm. The calibration curve was obtained using known concentrations of standard solutions purchased from Sigma Chemical Co.

### Quantification of Thiols

To measure extracellular thiols, H157 cells (7.0×10^5^) were seeded in 25 cm^2^ culture flasks from Sarstedts and incubated for 20 h. Cells were pretreated with MSG for 30 min, followed by treatment with +/− SAM and incubated for 5 h. To estimate intracellular thiols, H157 cells (2.0×10^5^/well) were seeded in a six-well plate for 24 h with subsequent treatment for 5 h with selenite or GS-Se-SG, +/− SAM. The thiol content was measured as described previously by the DTNB assay [40], [41].

### Viability Assay

In these series of experiments, cells were seeded at a density of 10 000 cells per well in 96-well plate. After 20 h incubation, cells were washed with PBS. Subsequently these were pre-treated with DTNB (50 µM) and MSG (60 mM) for 30 min, followed by treatment with the IC_50_ concentration of selenite (5 µM), GS-Se-SG (5 µM) and seleno-DL-cystine (100 µM) either in the presence or absence of SAM (500 µM, non-toxic dose) in a final volume of 100 µl. Viability was measured after 24 h by using XTT (Roche) labeling reagent at 470 nm and 650 nm using a spectrophotometer (Power Wave HT, BioTek).

To further study the toxicity over time, 7.0×10^5^ cells were seeded in 25 cm^2^ culture flasks in 10 ml growth medium and incubated for 20 h. Cells were washed with PBS and treated in the same manner as described above. Cells were trypsinized and counted at 0 h, 4 h, 8 h, 24 h, 30 h and 48 h. To compare the toxicity of the treatment with selenite +/− SAM and MSA, cells were seeded in 25 cm^2^ culture flasks as described above. Cells were treated for 24 h with MSA (5 µM), selenite (5 µM) +/− SAM (500 µM), harvested and counted.

### Clonogenic Assay

200 000 cells were seeded in petri dishes (6 cm^2^) and incubated overnight. Afterward, cells were treated with SAM (500 µM) and incubated for 8 h. Cells were washed with PBS, harvested and aliquots of 500 cells were re-seeded in growth medium in triplicates for 9 days. The colonies were fixed and stained with a crystal violet solution in acetic acid (50%) and ethanol (90%). Colonies were counted, and colonies of fewer than 50 cells were discarded. The efficiency of clonal growth was calculated by computing the ratio between the number of colonies formed and the number of cells seeded.

### Measurement of Superoxide-production

The production of superoxide was measured in H157 cells. Briefly, cells were seeded at a density of 7.0×10^5^ in 10 ml grown for 20 h in 25 cm^2^ flasks. Before treatment, cells were washed twice with PBS and incubated with 0.05 µM hydroethidine for 10 min in the dark. Thereafter, cells were washed three times with PBS and incubated for 5 h with MSA, selenite and selenite+SAM in RPMI 1640 without phenol red. Cells were harvested, washed twice with PBS and placed on ice. Fluorescence signal was analysed with a FACSCalibur II (BD Biosciences, Bedford, MA, USA) flow cytometer with an argon laser. Results were analysed using FlowJo 7 software for Windows (Tree Star Inc., Ashland, OR, USA).

### Statistical Analysis

All statistical analyses are based on at least three independent experiments and presented as mean±S.E.M. Statistical analysis of the protein disulfide reductase activity experiments were calculated using t-test, dependent by samples. The statistical method one-way ANOVA, followed by Tukey-Kramer multiple comparison test was used for calculation of uptake experiments, measurements of intracellular and extracellular thiols, and cell viability experiments. To determine the significance of the cell proliferation experiment (0–48 h), two-way ANOVA was performed, followed by Bonferroni multiple comparison test.

## Results

### SAM Mediated Formation of Methylated Selenium Compounds during Reduction of Selenite by the Thioredoxin and Glutaredoxin Systems

In an attempt to identify the methylated selenium species generated spontaneously upon an anaerobic reaction between selenite, SAM and the thioredoxin system, silver precipitation technique was used. The formation of methylated selenium species from the silver precipitate was confirmed by LDI-MS as methylselenol (Fig. 2B). Although superimposed with more abundant Ag_2_-containing clusters (see peaks at 310.8, 312.8 and 314.8 m/z), the spectrum of the precipitate showed the presence of [Ag_2_SeCH_3_]^+^, as assessed by the experimental pattern centered in the range 304.8–314.8 m/z. In particular, low-intensity peaks at 305.8, 307.8 and 309.8 m/z (highlighted with arrows in figure 2B) denoted the presence of selenium in the molecule. These peaks were not detected in the absence of thioredoxin (Fig. 2A). The pattern shown by [Ag_2_SeCH_3_]^+^ includes all the peaks reported for a synthetic [Ag_2_SeCH_3_]^+^ reference sample [37], and thus confirms the occurrence of a spontaneous and non-enzymatic methylation of selenide by SAM.

### Reduction of Selenite and GS-Se-SG by the Thioredoxin and the Glutaredoxin Systems in Presence of SAM

Selenite and GS-Se-SG are known to interact with the thioredoxin and glutaredoxin systems, resulting in non-stoichiometric oxidation of NADPH (6, 8, 39, 40). In order to investigate methylated selenium compounds as potential substrates for these systems, SAM was added to the reactions containing the selenium compounds. As expected, SAM alone was shown not to be a substrate for neither the thioredoxin nor the glutaredoxin system. However, addition of 4 mM SAM in the presence of one of the two selenium compounds increased the reaction rate by more than 3-fold (Fig. 3A–D). Under anaerobic conditions, the reaction of selenite with the thioredoxin system was slow and there was no effect on the reaction rate in the presence of SAM (data not shown). To measure the relative contribution of TrxR1 or Trx1 in the observed effect, the reactions were also performed in the absence of Trx1. The rate of NADPH oxidation was markedly reduced in the absence of Trx1. The absence of Grx1 almost abolished the NADPH consumption, demonstrating a negligible background reaction from glutathione. This observation shows that monomethylselenol is a superior substrate in redox cycles with O_2_ and the thioredoxin and glutaredoxin systems, compared to selenite/GS-Se-SG or selenide. However, the same reaction with seleno-DL-cystine was not affected by the presence of SAM (data not shown).

### Hydroperoxide Reduction by the Thioredoxin and Glutaredoxin Systems in the Presence of SAM

Although very moderate, the thioredoxin system reduces hydroperoxides, and the reaction rate is facilitated by selenium compounds [42]. The results in table 1 show a slow NADPH consumption with an expected rate by the inherent hydroperoxidase activity of the thioredoxin system, and SAM alone barely reacted with the thioredoxin system. The addition of selenite to the reaction confirmed the enhanced reaction rate, as previously reported [43]. The addition of SAM to this latter experiment with selenite resulted in a 3-fold increased rate of hydroperoxide reduction. Hydroperoxidase activity was also detected for the glutaredoxin system. The reaction rate was more than 2-fold higher than for the thioredoxin system and as for the thioredoxin system a 3-fold increase after the addition of SAM was observed (Table 2). The reduction of hydroperoxide was measured in the presence GS-Se-SG. This reaction was lower compared to the reaction with selenite. However, the reaction rate increased in a similar manner after the addition of SAM to the mixture. Seleno-DL-cystine, with inherent peroxidase activity, barely affected the reaction the presence of SAM (data not shown).

### The Effect of SAM on Selenite Mediated Inhibition of Protein Disulfide Reduction in the Presence of the Thioredoxin System

Selenite is a powerful inhibitor of insulin-disulfide reduction by the thioredoxin system [6]. The mechanism of inhibition is due to efficient NADPH oxidation and oxidation of thiols by selenium intermediates followed by ROS production. In the presence of 5 µM selenite the reduction of protein disulfides was inhibited by 35% (Fig. 4), while the presence of SAM resulted in a much stronger inhibition (65%). This data again show that methylselenol reacts more efficiently with the thioredoxin system and O_2_.

### Reduction of Cytochrome C by Monomethylselenol

Cytochrome C is known to be reduced by superoxide and selenide generated during interaction of selenite and the thioredoxin system [6]. The presence of SAM increased the selenite dependent reduction of oxidized cytochrome C. The reaction was monitored for 4 minutes and SAM enhanced the rate of reaction instantly of Cytochrome C. After one minute, 4.642 nmole of cytochrome C was reduced in the presence of SAM compared to 0.982 nmole in the absence of SAM (under the conditions described in materials and methods). The data show the presence of a nucleophilic moiety i.e., –Se^−^ and confirms the higher nucleophilicity of methylated selenium (e.g. CH_3_Se^−^) compared to HSe^−^.

### Selenium Uptake and Cellular Thiol Status

To explore the increased nucleophilicity and thus the reactivity of the methylated selenium compounds, selenium uptake and total thiol contents were determined in H-157 cells (Fig. 5A–D). To explore whether the uptake of the methylated selenium compounds differs from selenite and GS-Se-SG, cells were treated with MSG (a nontoxic concentration of 60 mM, [40]). Addition of MSG inhibits the X_c_^¯^ antiporter, by blocking the cystine uptake and consequently resulting in an oxidized extracellular environment. MSG pre-treatment was followed by the addition of selenite (5 µM) or GS-Se-SG (5 µM), at IC_50_ doses +/− SAM for 5 h. SAM was added in a nontoxic concentration (500 µM, defined by both clonogenic assay and XTT viability assay, Fig. 6A and 6D). Intracellular selenium accumulation was quantified by GF-AAS analysis (Fig. 5A and 5B) and revealed that SAM did not alter the cellular uptake of selenium after 5 h. In agreement with the previously reported data [40], pre-treatment of H157 cells with MSG lead to a strong inhibition of selenium uptake, which was sustained even after 5 h following the addition of SAM. To further exclude an interaction between SAM and MSG, the reduced extracellular thiol content was measured after 5 h of co-treatment with the two compounds, with no significant difference observed (Fig. 5C). Intracellular thiols were significantly decreased by selenite treatment and, interestingly, combination of these selenium compounds with SAM lead to an even more efficient decrease of intracellular sulfhydryls (Fig. 5D).

### Altered Cytotoxicity of Selenium Compounds in Cultured Cells in the Presence of SAM

The addition of SAM substantially increased the toxicity compared to selenite or GS-Se-SG alone, while SAM in combination with seleno-DL-cystine only exhibited a minor effect on the viability compared to single treatment of seleno-DL-cystine (Fig. 6A–C). To study the role of altered extracellular redox state or cystine uptake for the cytotoxic potential of selenium, cells were pre-incubated with MSG (60 mM) followed by a combined treatment with SAM (500 µM, nontoxic concentration, Fig. 6D) together with selenite (5 µM), GS-Se-SG (5 µM) or seleno-DL-cystine (100 µM) for 24 h. Blockage of cystine uptake with MSG, and consequently also selenium uptake, in the form of selenide, did not inhibit the cytotoxic effects of selenite or GS-Se-SG in combination with SAM.

To further explore the effects of MSG on the cytotoxicity, a prolonged study over time (48 h) was performed (Fig. 6E). MSG alone had no effect on cell proliferation during the 48 h treatment. Pre-treatment with MSG inhibited the toxicity of selenite over the 48 h of treatment, verifying a continuous blockage of cystine uptake. In the presence of SAM, the protective effect of MSG was only observed during the first 8 hours and was followed by significant cell death after 24 h of treatment (Fig. 6E). The cell death was consistent with the selenium uptake, which after treatment with SAM, selenite and MSG was initially (after 5 h) blocked (Fig. 5A), while a clear selenium accumulation was observed after 24 h of incubation (Fig, 6F). The results thus demonstrate that the delayed cytotoxicity could partly be explained by the delayed selenium accumulation. However, the increased cytotoxicity with the addition of SAM to selenite could not be explained by higher selenium uptake as this did not differ between the two treatments (Fig. 6D).

In an attempt to verify if the increased cytotoxicity, observed after addition of SAM, could be explained by formation of methylselenol, the cytotoxicity was further compared to treatment with MSA. As illustrated in Fig. 6G, MSA had a similar cytotoxic effect as selenite and SAM, and it was significantly higher than for selenite alone. Differences in morphological response are known to be elicited by MSA and selenite. Extensive cytoplasmic vacuolization with rounded cell shape was observed with selenite treatment, while in MSA treatment, attached cells exhibited elongated shape without such vacuolated structures [44]. However, cells displayed both of these common features in combined exposure to SAM and selenite (Fig. 6H).

### Intracellular Production of Superoxide

To further study the role of SAM in selenium mediated cytotoxicity, superoxide production was measured. The generation of superoxide was detected using hydroethidine in H157 cells after exposure to selenite in presence or absence of SAM. The results obtained clearly revealed the ability of selenite to increase basal level of cellular superoxide production. SAM co-treatment decreased the efficacy of selenite to enhance superoxide production by 50% after subtraction of the control (Fig. 7). As previously described by others, MSA did not generate any superoxide [45], [46], [47].

## Discussion

This paper reports novel interactions between SAM and selenium compounds in the presence of the two major redox systems, the thioredoxin and the glutaredoxin. SAM is a very reactive naturally occurring methyl donor that methylates a wide range of moieties in the cell in the presence of methyl transferases [34]. SAM may inhibit cancer cells growth by reversing hypo-methylated c-myc and H-ras in gastric and colon cancer [48]. Sulphur-bound methyl group in this molecule inherits high transfer potential, and may therefore also spontaneously methylate nucleic acids and proteins intracellularly [49] and selenide, as reported herein. In the presence of SAM, the kinetics of the well-established non-stoichiometric reactions of selenite/GS-Se-SG and the thioredoxin and glutaredoxin systems changed with a three-fold increased velocity aerobically. We have previously reported that the reduction of GS-Se-SG by the thioredoxin system is more efficient compared to the glutaredoxin system, while selenite is more readily reduced by the glutaredoxin system [8], [9]. This was also demonstrated in the presence of SAM in these reactions. There was no reaction with SAM in the absence of the tested selenium compounds, indicating a chemical modification of selenide in the presence of SAM (Fig. 8).

By LDI-MS spectrometry, we identified monomethylselenol as the active metabolite. The analysis was performed in a positive mode and therefore we cannot exclude the presence of other methylated forms in addition to monomethylselenol. Monomethylselenol is very unstable and volatile and therefore difficult to detect. However, we preserved the molecule under strict anaerobic conditions and precipitated a stable salt by silver prior to analysis. Monomethylselenol has previously been assumed to be formed from selenide by methyltransferases, from selenomethylselenocysteine by β-lyase or possibly in a very slow rate from selenomethionine catalyzed by γ-lyase in mammals. In other species monomethylselenol may be formed from SeMet as exemplified by methionine gamma lyase in fish [50] or by cystathionine gamma lyase in *S. cerevisiae* [51]. However, our data indicate that an efficient spontaneous methylation reaction occurs. The reaction requires the formation of the highly reactive reduced selenium anion formed by thioredoxin- or glutaredoxin-mediated reduction. Upon methylation, the selenium anionic moiety will be more nucleophilic and thereby more reactive. This increased nucleophilicity explains the higher aerobic reaction rate with the thioredoxin and glutaredoxin systems, the increased peroxidase activity and the more efficient reduction of cytochrome C. This property also complicates the interpretation of the data concerning ROS-formation. In the presence of sufficient reducing capacity, monomethylselenol will scavenge ROS due to the superior nucleophilicity compared to selenide, thereby leading to false low values in the ROS detection experiments as reported here and by others previously. Consistent with these interpretations, a diminished superoxide production is observed after treatment with selenite in the presence of SAM. Within the cell, monomethylselenol is a very unstable intermediate that may be demethylated to selenide or further methylated to the primary excretory product trimethylselenononium [52].

Monomethylselenol is considered to be a superior anti-cancer agent implicated in inducing apoptosis in cancer cells [53]. The mode of cell death induced by this intermediate differs compared to non-methylated species. Methylated species induce caspase-dependent apoptosis while inorganic forms e.g., selenide instead induce DNA-strand breaks, phosphorylation of p53 and caspase-independent apoptosis [47], [54], [55]. The cytotoxicity of selenite and GS-Se-SG increased in the presence of SAM. It is important to note that the uptake was essentially unaltered in the presence of SAM and thereby excluding the enhanced intracellular accumulation to be the mechanism of increased cytotoxicity. These observations led us to deduce that in the presence of SAM, the intermediate selenium compound formed is much more toxic than those of the intermediate metabolites of selenite and GSSeSG alone. Most likely this reflects the formations of extracellular methylated selenium species. Our results are in agreement with previously published data showing a superior cytotoxicity of the methylated selenium species compared to non-methylated forms [53]. In addition, our results revealed that the addition of SAM to selenite decreased the superoxide formation and altered the morphologically determined cell death when compared to selenite treatment alone and instead resembled that of MSA treated cells, which further corroborate with our observation on the formation of methylselenol.

We have previously reported that the cytotoxicity of selenite is dependent on the extracellular environment and that a reducing environment leading to extracellular reduction facilitates uptake [40]. The major extracellular reductant is cysteine and the reduction is dependent on a redox cycle with the uptake of cystine by the X_c_^¯^ antiporter. Our data from the present study revealed that the extracellular environment was not affected by SAM. The presence of MSG inhibited the cystine uptake and thereby protected the cells from selenite and GS-Se-SG induced cytotoxicity. Nevertheless, in the presence of SAM, a different pattern was observed. Initially the cells were protected by MSG but there was a delayed cytotoxic effect observed after 24 h. Our data showed that SAM did not interact with MSG, but several alternative considerations might explain the effect. Spontaneous methylation of cell surface structures including the X_c_^¯^ antiporter resulting in modulation of the permeability for cystine than a blockage of selenium uptake is one possible explanation. In addition, the formation of methylselenol under more oxidative conditions will be hindered leading to a delayed uptake of methylselenol.

In the presence of MSG, the combination of SAM and GS-Se-SG showed an enhanced cytotoxicity. This may indicate the formation of methylated glutathione selenopersulphide, GS-Se-CH_3_. This compound can only be stable in an oxidizing environment which MSG will provide. Neither the kinetics nor the cytotoxicity was altered in the experiments with seleno-DL-cystine in the presence of SAM. Spontaneous chemical modifications of selenide and the relatively higher reactivity and cytotoxicity of these species are of great physiological and pharmacological importance and provide mechanisms for the anti-tumor effects of selenium.
